# Annotations

**Published:** 1896-02-15

**Authors:** 


### Annotations.

### Why do not Women Insure their Lives?

WOMEN, at any rate in this country, do not insure their lives to anything like the same extent as men. It is, obvious, however, that as they assume more and more the *rôle* of breadwinners it will become necessary for them to make provision for those dependent upon them, exactly as men do. In America things are a little different in this respect. Women there insure much more largely, and not only insure, but take upon themselves the responsibilties of insurance work in various departments. Here, it seems to us, is a possible field for the energies of the enterprising among the gentler sex. Miss Emily A. Ransom, writing in the *Detroit Indicator*, goes pretty fully into the whole question of the insurance of female lives, and argues that if women are to be insured, as they should be, in largely-increased numbers, the result can only be brought about by employing female "solicitors," that is agents, to bring in the business, and female doctors to examine the pro posers when they have been brought in. There is a great deal to be said for these contentions. Why do not a syndicate of women open a life office and "run" it on their own account? A successful life office, started and "run" by women, would go far to demonstrate their fitness for at least one more of the high avocations usually given over to men. That women can and will insure, when the value of insurance is properly set before them, is proved by the unparalleled success of the Royal National Pension Fund.

### Who is to Judge?

THE widely different points of view from which social questions are regarded by those who study humanity in bulk and those who concern themselves only with individuals, is well illustrated by the fact that while philanthropists are everywhere engaged in the erection of institutions for the alleviation of individual suffering, those who interest themselves in "humanity," meaning thereby the progress of the race, do not hesitate to advocate a lethal chamber for the unfit, as a means of ridding the world of those who are useless members of society and likely, if they become parents, to cause degeneration of the race. When proposals of this nature are put forward "in all seriousness and reverence," we may well ask, Who is to judge? The medical profession has had many duties put upon it, and no doubt those who advocate such proceedings as are here alluded to would consider it a small matter to refer the decision as to fitness to a medical court. We think, however, that the medical profession would be as unwilling to accept such an office as the public would be to grant such power to any body of men in whatever way they might be selected. However widely medical men may interest themselves in matters of evolution and sociology, the whole tendency of their training is to make them feel tied in duty and in honour to the individuals entrusted to their care, and every medical man will be ready to declare that if once the question of "removal" rather than of cure were allowed to enter into consideration, it would open the floodgates to evils of the vastest and most horrible description; while we need only consider popular pronouncement in regard to our present somewhat feeble lunacy laws, to see that, so far as the public is concerned, no such power will ever be delegated. The proper study of heredity no doubt gives the key to a great deal which has appeared obscure in the evolution of the powers and attributes of man, and it is not difficult, in the light thus shed upon these questions, to see that, much in the same way as the breed of animals living in a state of nature is kept up by the destruction of the less perfect specimens, and by that process of sexual selection, or in other words fighting for mates, which makes the propagation of the race fall to the lot of the most fit, so in proportion as such influences as these are removed in the case of man, man tends to deteriorate. But the argument is unsound which would jump from that admission to the conclusion that, without undergoing these natural ordeals of combat for wives, and starvation of all who are not self-supporting, man can by a mere exercise of judgment eliminate the unfit. It is to be feared that we are as yet far from being wise enough to be able to say without chance of error, as it is suggested we should say, who are so unfit as to deserve to be deprived of the power of becoming parents, and who are so utterly unfit as to be worthy only of the lethal chamber. Over-shadowing the whole problem hangs, and always will hang, the question, Who is to judge?

### Fifteen Reasons for Vaccination.

THE medical profession is taking a leaf out of the book of the election agent, and striving to convince the reason of the public rather than to stand too strenuously upon legislative enactments in its pursuit of the physical well-being of the people. Dr. Thomas Bond, medical officer of health for Gloucestershire, sends us an excellent leaflet, which sets forth fifteen sober and convincing reasons in favour of general vaccination. We cannot give them all, but two samples will show the quality of the mass. The first is the homeliest of all reasons, and might have been expressed by the proverb that the "proof of the pudding is in the eating." For a century now, says Dr. Bond, vaccination has been in increasingly general use, and the result—or, at any rate, the fact—is that small-pox to-day is no more feared than gout. Another of the fifteen reasons is taken from the great Bishop Butler's logical armamentarium. "Probability," said Butler, "is the guide of life." He who in this world insists upon absolute certainty before he will take a step will never take a step at all. In like manner, Dr. Bond insists that since small-pox has so largely diminished wherever vaccination has prevailed, since it has not diminished in a like ratio among unvaccinated peoples; since it is now no longer a scourge, but almost a pathological curiosity, where vaccination is repeated at intervals; and since the medical profession, notwithstanding its enormous strides in science and learning during the past half-century, still believes in vaccination almost to a man, the probability of the value of the operation is so great that it practically amounts to a certainty. These two reasons are excellent. The remaining thirteen are all more or less good. The leaflet is short and interesting, and ought to be circulated by thousands.

